# Identification of Two Major QTLs in *Brassica napus* Lines With Introgressed Clubroot Resistance From Turnip Cultivar ECD01

**DOI:** 10.3389/fpls.2021.785989

**Published:** 2022-01-12

**Authors:** Fengqun Yu, Yan Zhang, Jinghe Wang, Qilin Chen, Md. Masud Karim, Bruce D. Gossen, Gary Peng

**Affiliations:** Saskatoon Research and Development Centre, Agriculture and Agri-Food Canada, Saskatoon, SK, Canada

**Keywords:** *Brassica napus*, *Brassica rapa*, *Plasmodiophora brassicae*, clubroot, genotyping by sequencing, ECD01, resistance, pathotype

## Abstract

*Plasmodiophora brassicae* causes clubroot disease in brassica crops worldwide. *Brassica rapa*, a progenitor of *Brassica napus* (canola), possesses important sources for resistance to clubroot. A doubled haploid (DH) population consisting of 84 DH lines were developed from a Backcross2 (BC_2_) plant through an interspecific cross of *B. rapa* turnip cv. ECD01 (resistant, R) with canola line DH16516 (susceptible, S) and then backcrossed with DH16516 as the recurrent parent. The DH lines and their parental lines were tested for resistance to four major pathotypes (3A, 3D, 3H, and 5X) of *P. brassicae* identified from canola. The R:S segregation ratio for pathotype 3A was 1:3, and 3:1 for pathotypes 3D, 3H, and 5X. From genotyping by sequencing (GBS), a total of 355.3 M short reads were obtained from the 84 DH lines, ranging from 0.81 to 11.67 M sequences per line. The short reads were aligned into the A-genome of *B. napus* “Darmor-*bzh*” version 4.1 with a total of 260 non-redundant single-nucleotide polymorphism (SNP) sites. Two quantitative trait loci (QTLs), *Rcr10^ECD01^* and *Rcr9^ECD01^*, were detected for the pathotypes in chromosomes A03 and A08, respectively. *Rcr10^ECD01^* and *Rcr9^ECD01^* were responsible for resistance to 3A, 3D, and 3H, while only one QTL, *Rcr9^ECD01^*, was responsible for resistance to pathotype 5X. The logarithm of the odds (LOD) values, phenotypic variation explained (PVE), additive (Add) values, and confidence interval (CI) from the estimated QTL position varied with QTL, with a range of 5.2–12.2 for LOD, 16.2–43.3% for PVE, 14.3–25.4 for Add, and 1.5–12.0 cM for CI. The presence of the QTLs on the chromosomes was confirmed through the identification of the percentage of polymorphic variants using bulked-segregant analysis. There was one gene encoding a disease resistance protein and 24 genes encoding proteins with function related to plant defense response in the *Rcr10^ECD01^* target region. In the *Rcr9^ECD01^* region, two genes encoded disease resistance proteins and 10 genes encoded with defense-related function. The target regions for *Rcr10^ECD01^* and *Rcr9^ECD01^* in *B. napus* were homologous to the 11.0–16.0 Mb interval of chromosome A03 and the 12.0–14.5 Mb interval of A08 in *B. rapa* “Chiifu” reference genome, respectively.

## Introduction

*Brassica* species are grown for the production of edible oil and vegetables. The genomic relationships among the main species of brassica crops were explained by the “triangle of U” (Morinaga, 1934; Nagaharu and Nagaharu, 1935); *Brassica rapa* (genome represented as AA; *n* = 10), *Brassica nigra* (BB; *n* = 8), and *Brassica oleracea* (CC; *n* = 9) are diploid species, and *Brassica napus* (AACC; *n* = 19), *Brassica juncea* (AABB; *n* = 18), and *Brassica carinata* (BBCC; *n* = 17) are amphidiploid species resulting from hybridization between pairs of the diploid species.

Clubroot, caused by the obligate soil-borne pathogen *Plasmodiophora brassicae* Woronin, is an important disease in brassica crops worldwide. The pathogen belongs to the infrakingdom Rhizaria, a diverse group of amoeboid microbes (Nikolaev et al., 2004). Root infection by *P. brassicae* results in the formation of characteristic clubs, also known as “galls,” on the roots of host plants. These abnormal growths restrict the flow of water and nutrients to the plant, resulting in above-ground symptoms that include stunting, yellowing, premature senescence, and reduction in both seed yield and quality (Pageau et al., 2006). *B. napus* (oilseed rape/canola) is an important crop for edible oil production worldwide. Clubroot was first identified in canola fields on the Canadian Prairies in 2003 but has spread rapidly to pose a serious threat to canola production in Canada.

Strains of *P. brassicae* collected in Canada have been classified into more than 30 pathotypes based on the reactions on the Canadian Clubroot Differential (CCD) set (Strelkov et al., 2018; Hollman et al., 2021). Among the pathotypes, 3H was the most prevalent original pathotype, 5X was the first new pathotype that was aggressive on the first generation of Canadian clubroot-resistant cultivars, and 3A and 3D are currently the predominate new pathotypes in Alberta (Dakouri et al., 2021). The pathogen can survive in soil as resting spores for a long period, so it is difficult to manage using cultural practices or chemical treatments (Voorrips, 1995). Genetic resistance can be an effective strategy for clubroot management, but the sources available for resistance to clubroot in *B. napus* are very limited. Strong resistance was identified in its progenitor species, *B. rapa*, especially in European turnip, *B. rapa* subsp. *rapifera*, which was reviewed by Hirai (2006). The resistance to clubroot available from European turnips has been transferred into Chinese cabbage (*B. rapa*) (Piao et al., 2009). Introgression of traits from turnip into *B. napus* is possible *via* interspecific crosses, so turnip has been a valuable source for resistance to clubroot in canola. Clubroot resistance (CR) from turnip cultivar “Debra” has been transferred into *B. napus* cultivars of swede (Lammerink, 1970) and from turnip cultivar “Waaslander” (also known as ECD04) into forage (Johnston, 1974) and oilseed lines of *B. napus* (Gowers, 1982).

Genetic mapping of CR genes is an important step toward breeding for resistance to clubroot. To date, more than 20 genes or quantitative trait locus (QTLs) have been mapped to six chromosomes of the A-genome in *B. rapa* through biparental mapping methods. *Crr2* and *PbBa1.1* were located on A01 (Suwabe et al., 2003; Chen et al., 2013); *CRc* and *Rcr8* were located on A02 (Sakamoto et al., 2008; Yu et al., 2017); *Bra.CR.a*, *Bra.CR.c*, *Crr3*, *CRa, CRb*, *CRb^kato^*, *CRd, CRk*, *PbBa3.1*, *PbBa3.2, PbBa3.3*, *Rcr1*, *Rcr2*, *Rcr4*, and *Rcr5* were located on A03 (Matsumoto et al., 1998; Hirai et al., 2004; Piao et al., 2004; Sakamoto et al., 2008; Chen et al., 2013; Chu et al., 2014; Pang et al., 2014; Yu et al., 2016, 2017; Huang et al., 2017, 2019; Hirani et al., 2018); *Crr4* were located on A06 (Suwabe et al., 2003); *qBrCR38-1* were located on A07 (Zhu et al., 2019); *Crr1, CRs, PbBa8.1*, *Bra.CR.b, Rcr3*, *Rcr9/Rcr9*^wa^**, and *qBrCR38-2* were located on A08 (Suwabe et al., 2006; Chen et al., 2013; Yu et al., 2017; Hirani et al., 2018; Laila et al., 2019; Zhu et al., 2019; Karim et al., 2020). Three genes, *Crr1*, *CRa*, and *CRb^kato^*, have been cloned, all of which encode toll-interleukin-1 receptor, nucleotide binding site, and leucine-rich repeat (TIR-NBS-LRR, TNL) proteins (Ueno et al., 2012; Hatakeyama et al., 2013, 2017). The identification of CR genes has been also carried out in *B. oleracea* (Lee et al., 2016; Dakouri et al., 2018; Peng et al., 2018), *B. nigra* (Chang et al., 2019), and *B. napus* (Manzanares-Dauleux et al., 2000; Werner et al., 2008; Fredua-Agyeman and Rahman, 2016; Hasan and Rahman, 2016; Botero-Ramírez et al., 2020).

*Brassica rapa* turnip cv. “Debra” was used as the donor for developing CR in swede cultivars (Lammerink, 1970). “Debra” was included in the European clubroot differential (ECD) set as differential line ECD01 (Buczacki et al., 1975; Diederichsen et al., 2009). *CRb*, a CR gene identified in a Chinese cabbage cv. “CR Shinki” and two CR genes in Chinese cabbage cv. “CR Kanko,” *CRk* and *CRc*, were derived from ECD01 (Piao et al., 2004) and “Debra” (Sakamoto et al., 2008), respectively. Two other CR genes, *BraA.CR.a* (A03) and *BraA.CR.b* (A08), were also identified from ECD01 (Hirani et al., 2018). Finally, ECD01 was resistant to all of the Canadian pathotypes of *P. brassicae* described by Strelkov et al. (2018) (Yu F, unpublished data), which makes it a valuable source of genes for CR canola in Canada.

In this study, an interspecific cross of ECD01 × *B. napus* line DH16516 was made, and the resulting F_1_ progeny were backcrossed with DH16516 to produce BC_1_. Continuing backcross was made by crossing the BC_1_ with DH16516 to produce Backcross2 (BC_2_). A doubled haploid (DH) population consisting of 84 DH lines from a single BC_2_ plant was developed. Genotyping by sequencing (GBS) analysis of the A-genome of *B. napus* was used to (1) characterize the genome-wide DNA variants in the DH lines, (2) detect QTLs associated with resistance to the most important pathotypes of *P. brassicae* on the Canadian Prairies, and (3) identify putative candidate genes for each QTL.

## Materials and Methods

### Plant Materials

A seed of ECD01, a turnip (*B. rapa*) cultivar carrying genes for CR, was provided by Nutrien Ag Solutions (Saskatoon, SK, Canada). DH16516 is a spring-type, clubroot-susceptible, DH canola-quality line of *B. napus* developed by Dr. Séguin-Swartz at Saskatoon Research and Development Centre, Agriculture and Agri-Food Canada (SRDC, AAFC), Saskatoon, SK, Canada. ECD01 was crossed to DH16516 (pollen donor) to produce F_1_ progeny. Backcrosses with DH16516 (recurrent parent) were performed to produce the BC_1_ and BC_2_ populations. A BC_2_ plant with resistance to pathotype 5X was chosen as the donor for microspore culture. In total, 84 DH lines were developed by Haplotech Inc. (Winnipeg, Canada) through a fee-for-service contract. Seed from three plants of each DH line was increased in a greenhouse at SRDC for study.

### Evaluation of Resistance to Clubroot

Clubroot strains collected in canola fields in Alberta were characterized based on pathotype and provided by Dr. S. E. Strelkov at the University of Alberta, Canada. The method and experimental design used in this study were as described by Suwabe et al. (2003). Plants were tested for resistance to four pathotypes of *P. brassicae* (strain F.3-14 for pathotype 3A, F.1-14 for 3D, P. 41-14 for 3H, and LG02 for 5X). Fresh and clean clubbed roots harvested at 4–5 weeks after inoculation of each strain were cut into smaller pieces with scissors, macerated in distilled water for 1–2 h, and blended in a blender at high speed for 2 min. After filtering through eight layers of sterile cheesecloth, resting spores extracted from the clubbed roots were adjusted to a concentration of 1.0 × 10^7^ resting spores/ml in distilled water for plant inoculation.

Seeds of the DH population were sown into Sunshine #3 soilless mix (Sun Gro Horticulture Canada Ltd., Seba Beach, AB, Canada) with Osmocote (Everris NA Inc., Dublin, OH, United States) in 32 pot inserts held by trays (The HC Companies, Twinsburg, OH, United States). Approximately 4 L of water was added to each tray to soak the soilless mix overnight. Seven days after planting, inoculation was performed by adding 15 ml of inoculum (1 × 10^7^ spores/ml) into each pot with 6–9 seedlings of each line. The inoculated plants were grown in a growth chamber set at 22/18°C day/night temperature with a 16-h photoperiod. The canola cultivar “45H29” (resistant to pathotype 3H) and the parental lines (ECD01 and DH16516) were included as controls. Six weeks after inoculation, plants were pulled and the roots were examined for clubroot symptoms.

Clubroot severity was evaluated on a 0–3 scale, where 0 indicates no clubbing, 1 indicates a few small clubs, 2 indicates moderate clubbing, and 3 indicates severe clubbing. A disease severity index (DSI) was calculated for each line using the method of Horiuchi and Hori (1980):

$$\text{DSI}=\frac{\sum \,\left(\mathrm{rating}\,\mathrm{class}\right)\,\times \,\left(\#\,\mathrm{plants}\,\mathrm{in}\,\mathrm{rating}\,\mathrm{class}\right)\,}{\text{total}\,\#\,\mathrm{plants}\,\mathrm{in}\,\mathrm{treatment}\times \,3}\times 100$$

Correlation coefficients of severity among the DH families to four pathotypes of *P. brassicae* were calculated in Microsoft Excel function “*Correl*” using the equation:

$$C\,o\,r\,r\,e\,l\,\,\left(X,Y\right)=\,\frac{\sum \left(x-\bar{x}\right)\,\left(y-\bar{y}\right)}{\sqrt{\sum {\left(x-\bar{x}\right)}^{2}\,\sum {\left(y-\bar{y}\right)}^{2}}}$$

Significance was determined using *t*-tests (Iversen and Gergen, 1997). Each line with a resistance response (DSI ≤ 30%) in the initial study was reassessed two more times. Each of these repetitions provided a similar result in most cases. For those lines with inconsistent results, the highest DSI among the three repetitions of the assessment was considered to be the most accurate and was used to characterize the resistance response of the line. DH lines with DSI ≤ 30% were classified as R and those lines with DSI >30% as S lines.

The F_1_ plants were tested with pathotype 3H, and the BC_2_ donor plant was only tested with 5X for several reasons. First, the clubroot reaction of a single plant can be assessed for only one pathotype. Second, pathotype 3H was the predominant pathotype in Canada when we obtained the F_1_ progeny and performed the selection for CR. Similarly, at that time when the donor plant from BC_2_ was chosen for microspore culture, pathotype 5X was the only new pathotype that had been identified. Third, only a few seeds of F_1_ were obtained due to difficulties in the interspecific cross, so it was not possible to test multiple pathotypes in the F_1_ progeny.

### DNA Sequencing and Alignment of Reads to a Reference Genome

DNA was extracted from young leaves of each of the 84 DH lines and parental lines following the DNeasy Plant Mini Handbook from QIAGEN. GBS of the 84 DNA samples and two replications of the parental cultivar ECD01 were performed on an Illumina platform with pair-end sequencing at BGI Americas Corp (Cambridge, MA, United States). Two replications of cv. ECD01 were performed to increase the sequencing depth for this parental line to provide a more accurate call of the genotype at each single-nucleotide polymorphism (SNP) site in the DH population. DH16516 is an important *B. napus* canola recipient line for introgression of CR at AAFC, Saskatoon, so whole-genome sequencing of the line had already been performed at Plant Biotechnology Centre (Saskatoon, SK, Canada) as part of the generation of a new reference genome (unpublished data). The short reads from the whole-genome sequencing data were used for this study. The program SeqMan NGen 15 (DNASTAR, Madison, WI, United States) was used for short read assembly. ‘‘Whole genome DNA-Seq/Genotyping’’ assembly workflow, ‘‘Reference based assembly-normal workflows’’ assembly type, and ‘‘Automatic Mer size, Automatic Minimum match percentage, High Layout stringency and Medium SNP filtering stringency’’ assembly options were chosen. Short reads from each of the 84 DH samples, parental DH16516, and the combined two replicates of ECD01 were aligned to *B. napus* reference genome for cv. ‘‘Darmor-*bzh*’’ version 4^1^.

### Identification of Variants, Variant Filtering, Construction of Linkage Map, and Quantitative Trait Locus Mapping

Identification of variants (SNPs and InDels) in the DNA sequences of each BC_2_ DH sample relative to the reference genome of *B. napus* “Darmor-*bzh*” was performed using SeqMan Pro 15 (DNASTAR, Madison, WI, United States), but only SNPs were used for further study. Comparison of the variants among the 84 BC_2_ DH samples was carried out using Qseq 15 (DNASTAR).

Genotyping by sequencing-SNP sites were named based on the reference genome (DM: “Darmor-*bzh*”), the A-genome chromosome (A01–A10), and the position on the reference chromosome sequence. An SNP site was called in a given sample at following criteria: depth >5, *Q* > 30, and SNP percentage >50%. Since the recipient parent DH16516 and the 84 DH lines were DH lines, all SNP sites should theoretically be homozygous. After filtering, heterozygous genotypes in the parental line DH16516 and the DH lines and monomorphic phenotypes between the parents or among the 84 individuals were removed.

The remaining SNP sites after filtering were further analyzed using JoinMap 4.1 (Kyazma B.V.,Ln.v.A. Wageningen, Netherlands; Van Ooijen, 2011). SNP alleles from the resistant parent (ECD01) were scored as “B,” and those from the susceptible parent (DH16516) as “A.” Marker orders and positions in the genetic map were determined using maximum likelihood in the Kosambi’s model with a minimum logarithm of the odds (LOD) values of 10. Only SNP sites that could be assigned into the 10 chromosomes of the A-genome at LOD scores of 10.0 were kept. The set of filtered SNP sites obtained was used for binning of redundant markers, construction of linkage map, and mapping of QTLs for resistance to clubroot using the QTL IciMapping Inclusive Composite Interval Mapping (ICIM) method (Meng et al., 2015). A linkage map was drawn using MapChart 2.1 (Droevendaalsesteeg 4, Wageningen, Netherlands; Voorrips, 2002) based on the genetic location determined with QTL IciMapping. The LOD score threshold was set using a 1,000-permutation test with a type I error of 0.05 for QTL declaration. The QTL effects were estimated as phenotypic variation explained (PVE) and additive (Add) values by each QTL.

### Identification of Genes in the Target Regions of the *B. napus* “Darmor-*bzh*” Reference Genome

Gene annotation was analyzed using Blast2GO (Conesa et al., 2005) using coding sequences (CDS) of the genes in each of the QTL target regions from 1 Mb upstream to 1 Mb downstream of the SNP markers in the peak regions as determined by IciMapping. Genes related to disease resistance and defense responses were identified using Blast2GO information of the gene description and gene ontology. The most probable Arabidopsis homolog corresponding to each disease resistance gene and the class of disease resistance proteins were obtained using the CDS of the disease resistance gene in the *B. napus* by Blast search at www.arabidopsis.org.

### Mapping of the Quantitative Trait Loci With Bulked Segregant Analysis

Bulked segregant analysis (BSA) has been used to detect molecular markers linked to traits of interest, such as disease resistance (Michelmore et al., 1991). In BSA, bulks of plants with contrasting phenotypes are generated. Our previous studies showed that a gene could be genetically mapped by identifying the percentage of polymorphic variants (PPV) in a genome using BSA (Yu et al., 2016; Dakouri et al., 2018; Huang et al., 2019; Karim et al., 2020).

Doubled haploid lines were selected to form a R bulk and a S bulk based on their phenotypes using SNP marker-assisted selection. GBS data from the R and S bulks were aligned onto the *B. napus* reference genome separately using SeqMan NGen 15 (DNASTAR). Mapping of the QTLs was performed using the PPV method described by Yu et al. (2016) and Dakouri et al. (2018).

### Search for the Syntenic Regions of Identified Quantitative Trait Loci in *B. rapa* “Chiifu” Reference Genome

The *B. rapa* reference genome version 3.0 (Zhang et al., 2018) was downloaded from https://brassicadb.org/brad/downloadOverview.php. DNA sequences of the QTL target regions from the A-genome of *B. napus* were aligned into the *B. rapa* genome using MegAlign Pro 15 with MAUVE (DNASTAR).

## Results

### Resistance to Clubroot in the Parental Lines and the Backcross2 Doubled Haploid Population

**FIGURE 1:**
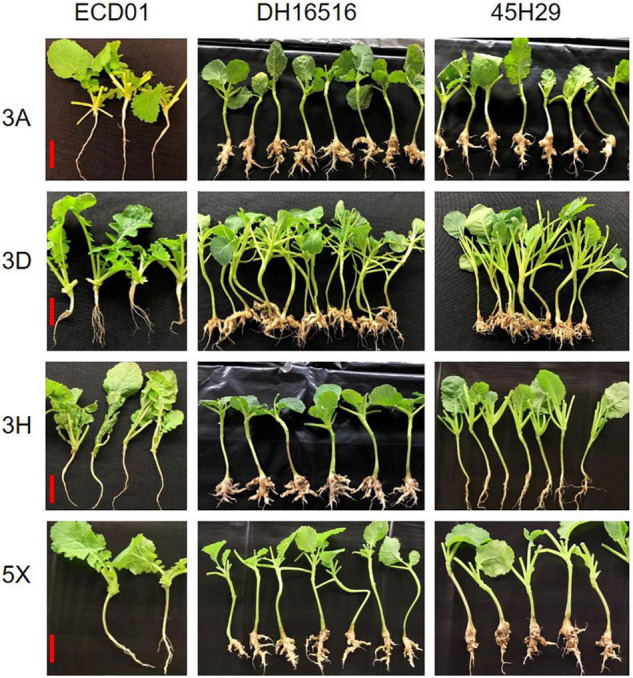
Plant phenotypes and clubroot response at 5 weeks after inoculation in the parental lines (Debra and DH16516) and a control cultivar (45H29) to inoculation with four pathotypes (3A, 3D, 3H, and 5X) of *Plasmodiophora brassicae* under controlled conditions. The bars represent 5 cm in length.

**FIGURE 2:**
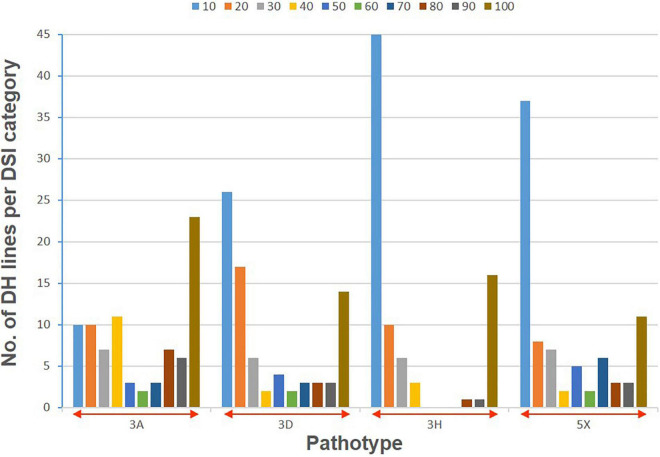
Distribution of clubroot severity (disease severity index, DSI) following inoculation with four pathotypes (3A, 3D, 3H, and 5X) of *P. brassicae* in a doubled haploid (DH) population derived from a BC_2_ plant of *Brassica rapa* ECD01 crossed with *B. napus* DH16516. Colors in each stacked column represent the proportion of the lines with a DSI value within that decile (= 10% range).

The clubroot reaction of the parental lines (ECD01 and DH16516), controls, and the DH population was assessed against pathotypes 3A, 3D, 3H, and 5X (Table 1). As expected, ECD01 was highly resistant to all pathotypes (0% DSI), DH16516 was highly susceptible (100% DSI), and “45H29” was resistant to pathotype 3H only (Figure 1 and Table 1). The F_1_ plants from the interspecific crosses of DH16516 × ECD01 were highly resistant to pathotype 3H (0% DSI), which was the predominate pathotype in Canada before the emergence of the 3A, 3D, and 5X. Clubroot severity in response to inoculation with each pathotype in the DH population could be divided into two classes: resistant (R) lines with DSI ≤ 30% and susceptible (S) lines with DSI > 30% (Figure 2). The segregation ratio of R and S was calculated, and the goodness-of-fit was tested with a χ^2^ test using Microsoft Excel software. Of the four pathotypes, segregation of R and S best fit a 1:3 ratio for pathotype 3A and a 3:1 ratio for pathotypes 3D, 3H, and 5X. These results indicated that resistance to pathotype 3A was controlled by two genes in complementary action, and resistance to pathotypes 3D, 3H, and 5X was controlled by two genes in duplicate action.

**TABLE 1:** Genetic analysis of resistance of the parental lines (DH16516, ECD01), controls (cv. “45H29”), and the inoculation of doubled haploid (DH) population derived from BC_2_ with four pathotypes of *Plasmodiophora brassicae* based on the clubroot severity (disease severity index, DSI) of each line (Resistant, R, DSI ≤ 30; Susceptible, S, DSI > 30).

Patho type	DSIs				No. of DH lines			*P*-value of ratio		
	ECD01	DH16516	F_1_	45H29	Total	R	S	1:1	3:1	1:3
3A	0	100	–	100	82	27	55	0.001	0.001	0.100
3D	0	100	–	100	80	49	31	0.001	0.005	0.001
3H	0	100	0	0	82	61	21	0.001	0.90	0.001
5X	0	100	–	100	84	52	32	0.001	0.006	0.001

Correlation coefficients among the DSI values for the pathotypes ranged from 0.55 to 0.81, but all were significant at *P* < 0.01 (Table 2). This indicated that the genes for resistance to the different pathotypes were likely controlled by the same genes or tightly linked genes.

**TABLE 2:** Correlation coefficients for clubroot severity after inoculation of DH population derived from BC_2_ of DH16516 × ECD01 for resistance to four pathotypes of *P. brassicae*.

Pathotype	3A	3D	3H	5X
3A	1.00			
3D	0.64**	1.00		
3H	0.65**	0.81**	1.00	
5X	0.55**	0.55**	0.68**	1.00

**Significance level at P < 0.01.

### Alignment of DNA Short Reads Into the *B. napus* Genome

Since CR in the DH population originated from the A-genome of *B. rapa* cv. ECD01, only A-genome DNA sequences in the reference genome *B. napus* “Darmor” version 4.1 were used for alignment of DNA short reads and discovery of DNA variants (SNPs and InDels). Approximately 219.9 million (M) short reads were obtained from whole-genome sequencing from DH16516, and 53.1% of the reads were assembled into the reference A-genome; 13.8 M sequences were obtained from GBS of ECD01, and 70.9% were assembled into the reference A-genome. A total of 355.3 M short reads from 84 DH lines were obtained, ranging from 0.81 to 11.67 M sequences per line (Supplementary Figure 1). The mean number of reads aligned into the reference genome from each line was 2.3 M (range 0.46–5.22 M, Supplementary Figure 1), and 54.7% were assembled into the reference A-genome.

### Identification of Polymorphic Single-Nucleotide Polymorphism Sites and Quantitative Trait Locus Analysis

After the initial filtering, 429 polymorphic SNP sites were left and were distributed to 9 of 10 chromosomes of the reference genome of “Darmor-*bzh*” (Supplementary Table 1). No polymorphic markers were identified from chromosome A06. There was no correlation between chromosome size and the number of SNP markers identified (*r* = −0.092) in the population. To remove redundant markers, the 429 SNP sites were further filtered using the binning function in IciMapping, which left only 260 non-redundant SNP sites (Table 3). A genetic map of the nine chromosomes of the A*-*genome was constructed from the distributed SNP sites (Supplementary Figure 2). The length of each chromosome ranged from 0 (chromosome A06) to 471.8 cM (A01), with an average length of 85.3 cM. Chromosome A01 was much longer than the other linkage groups. The number of SNP sites per chromosome ranged from 0 (A06) to 152 (A01), with a mean of 26 SNPs per chromosome. The SNP interval of each chromosome ranged from 0.8 to 4.8 cM, with a mean of 3.3 cM (Supplementary Table 1).

**TABLE 3:** QTL position, phenotypic variation explained (PVE), additive (Add), the logarithm of the odds (LOD), and confidence interval (CI) for the QTLs originating from *Brassica rapa* ECD01 for resistance to four pathotypes of *P. brassicae* (permutations = 1,000).

Pathotype	Chromosome/QTL	Position	Left marker	Right marker	LOD	PVE (%)	Add	Left CI	Right CI
3A	A03/*Rcr10*^ ECD01^	0	DM_A03_12570715	DM_A03_10873502	5.6	16.2	14.3	0.0	1.5
	A08/*Rcr9^ECD01^*	38	DM_A08_10325589	DM_A08_10529713	11.4	43.3	22.4	35.5	42.5
3D	A03/*Rcr10*^ ECD01^	0	DM_A03_12570715	DM_A03_10873502	7.2	27.3	21.4	0.0	2.5
	A08/*Rcr9^ECD01^*	38	DM_A08_10325589	DM_A08_10529713	5.2	21.5	18.1	32.5	44.5
3H	A03/*Rcr10*^ ECD01^	0	DM_A03_12570715	DM_A03_10873502	6.2	17.9	16.6	0	2.5
	A08/*Rcr9^ECD01^*	35	DM_A08_10337601	DM_A08_10325589	12.2	43.1	25.2	33.5	37.5
5X	A08/*Rcr9^ECD01^*	34	DM_A08_10337601	DM_A08_10325589	10.9	42.8	25.4	32.5	37.5

**FIGURE 3:**
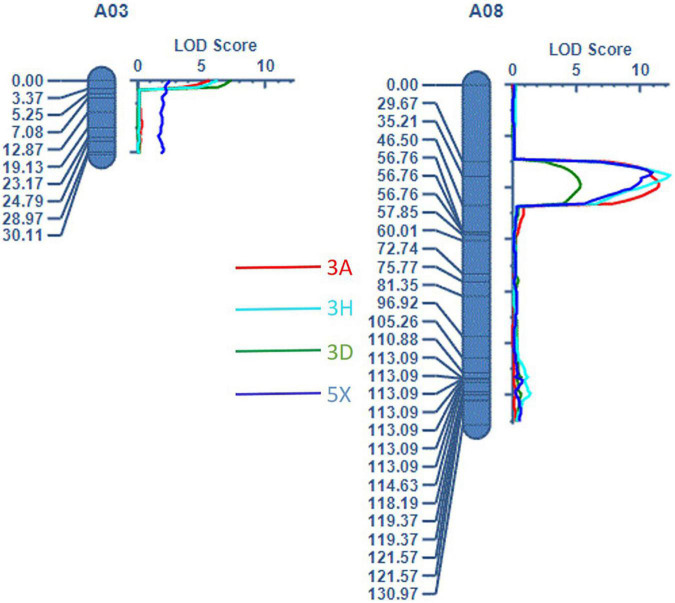
Two QTLs were detected: *Rcr10^ECD01^* on chromosome A03 and *Rcr9^ECD01^* on A08.

Mapping of the QTLs was performed using the linkage map (Supplementary Figure 1) and trait values for resistance to each pathotype (3A, 3D, 3H, and 5X). Two QTLs were identified: a QTL designated as *Rcr10^ECD01^* on A03, with a peak at the SNP markers DM_A03_12570715 and DM_A03_10873502, and a QTL designated as *Rcr9^ECD01^* (Figure 3), located near the previously identified genes *Rcr9* and *Rcr9^wa^* (Yu et al., 2017; Karim et al., 2020) on A08, with a peak at DM_A08_10325589 and DM_A08_10529713 (Table 3). Resistance to pathotypes 3A, 3D, and 3H was associated with the two QTLs (*Rcr10^ECD01^* and *Rcr9^ECD01^*), but resistance to 5X was only associated with *Rcr9^ECD01^* (Table 3). LOD, PVE, Add values, and CI from the estimated QTL position varied between the QTLs, ranging from 5.2 to 12.2 for LOD, 16.2 to 43.3% for PVE, 14.6 to 25.4 for Add, and 1.5 to 12 cM for CI (Table 3). The values of Add for the two QTLs were positive, indicating that the resistant loci were derived from the resistant parent ECD01.

### Identification of Disease Resistance Genes and Genes Related to Plant Defense Response

Searches for candidate genes for *Rcr10^ECD01^* and *Rcr9^ECD01^* that encoded disease resistance proteins and defense-related genes were performed using CDS of the reference genome in the target region including 1 Mb up- and downstream of the left and right markers (Table 3).

*Rcr10^ECD01^*, which was responsible for resistance to pathotypes 3A, 3D, and 3H, was mapped into chromosome A03, with a peak at SNP markers DM_A03_10873502 and DM_A03_12570715 (Table 3). There are 676 genes in this 3.7 Mb region (Supplementary Table 3). Among the genes, one gene (*BnaA03g25330D*) encoded a disease resistance protein (Table 4), and 24 genes encoded proteins with functions related to plant defense response (Supplementary Table 3). *BnaA03g25330D* is homologous to the Arabidopsis gene *AT5G22690*, which encoded a TNL protein (Table 4).

**TABLE 4:** A list of genes encoding proteins associated with plant disease resistance through BLAST2GO and Blast searches with CDS in the QTL target regions at https://www.arabidopsis.org/Blast/index.jsp.

QTL	*Rcr10^ECD01^*	*Rcr3/9^ECD01^*	
**R to pathotype**	3A, 3D, and 3H	3A, 3D, 3H, and 5X	
**Chromosome**	A03	A08	
**Gene name**	*BnaA03g25330D*	*BnaA08g10100D*	*BnaA08g11840D*
***B. napus* gene location (base)**	12234711…12240552	9456084…9467947	10622229…10625339
**Length (base)**	5841	11863	3110
**Description from Blast2GO**	Disease resistance protein RPS6-like	Disease resistance protein TAO1-like	Probable disease resistance protein At4g33300
**Function with Blast2GO**	Hydrolase activity; ADP binding; defense response; signal transduction	Hydrolase activity; ADP binding; defense response; signal transduction	ADP binding
**Homolog in Arabidopsis**	*AT5G22690*	*AT5G11250*	*AT4G33300*
**R gene class**	Disease resistance protein (TIR-NBS-LRR class) family	TIR-NBS-LRR protein involved in stress response	Activated disease resistance 1 (ADR1) family of NBS-LRR immune receptors

*Rcr9^ECD01^*, which was responsible for resistance to all four pathotypes, was mapped into chromosome A08, with a peak at SNP markers DM_A08_10325589 and DM_A08_10529713. There were 338 genes in this 2.2 Mb region (Table 4 and Supplementary Table 3). Two genes (*BnaA08g10100D* and *BnaA08g11840D*) encoded disease resistance proteins, and *BnaA08g10100D* was homologous to the previously cloned resistance gene *Crr1*. *BnaA08g10100D* and *BnaA08g11840D* were homologous to the Arabidopsis genes *AT5G11250* and *AT4G33300*, respectively. *AT5G11250* encodes an atypical TNL protein and *AT4G33300* encodes a member of the activated disease resistance 1 family nucleotide-binding leucine-rich repeat immune receptors (Table 4). Also, this region contained 10 genes that encoded proteins with defense-related functions (Supplementary Table 3).

### Confirming the Quantitative Trait Locus Intervals With Bulked Segregant Analysis

Of the 84 DH lines, 19 lines were resistant to almost all the pathotypes. They all carried alleles from the resistant parent ECD01 (SNP genotype “B”) with *Rcr10^ECD01^* (DM_A03_10873502 and DM_A03_12570715) and *Rcr9^ECD01^* (DM_A08_10325589 and DM_A08_10529713). Also, 17 lines were susceptible to almost all the pathotypes and all of them carried alleles from the susceptible parent line DH16516 (SNP genotype “A”) for the two QTLs. As a result, the R bulk was formed from the 19 R DH lines, while the S bulk was formed from the 17 S DH lines for the BSA (Supplementary Table 4).

**FIGURE 4:**
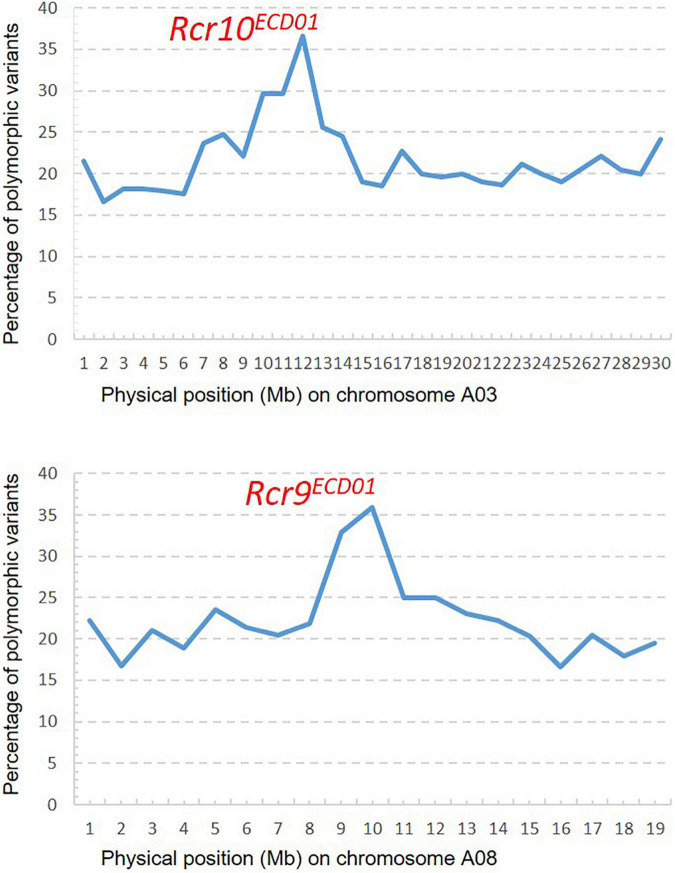
Distribution of polymorphic variants (%): One peak for *Rcr10^ECD01^* on chromosome A03 and the other for *Rcr9^ECD01^* on A08 were identified through bulk segregant analysis with the mapping method of the percentage of polymorphic variants described by Yu et al. (2016) and Dakouri et al. (2018).

A total of 93.5 M short reads from the R bulk and 69.4 M short reads from the S bulk were aligned into the *B. napus* reference genome. A PPV peak (25–30%) occurred within the physical interval 9–14 Mb on chromosome A03 and the other peak (25–36%) within the physical interval 9–12 Mb on chromosome A08 (Figure 4), which indicated that *Rcr10^ECD01^* and *Rcr9^ECD01^* resided in the intervals of chromosomes A03 and A08, respectively. This result is consistent with that from the above QTL analysis.

### Search for the Syntenic Regions of the Quantitative Trait Loci in the *B. rapa* “Chiifu” Reference Genome

Most of the genes or QTLs for CR in *Brassica* species containing the A-genome that have been identified were from *B. rapa*. In this study, the DH population was developed with introgression of QTLs from *B. rapa*, so the QTL target regions of chromosome A03 and A08 of *B. napus* were compared with those of *B. rapa*.

**FIGURE 5:**
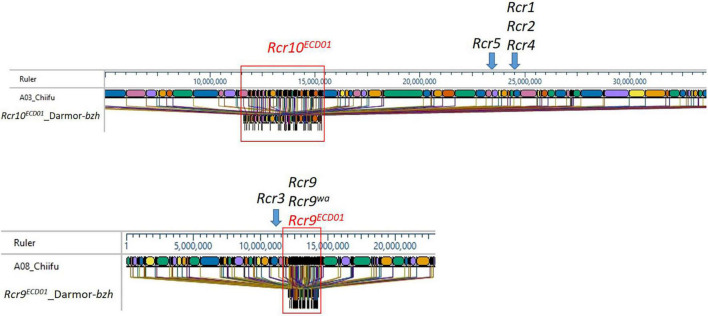
Maps of the *Rcr10^ECD01^* and *Rcr9^ECD01^* target regions in A03 and A08 of *B. napus* “Darmor” and the homologous regions of the *B. rapa* reference genome “Chiifu” version 3.0. Different colors represent the sequenced region of the *B. rapa* reference genome “Chiifu” version 3.0.

The *B. rapa* reference genome “Chiifu” version 3.0 is the most recent version available for the “Chiifu” reference genome (Zhang et al., 2018). The 3.7 Mb region from 9.8 to 13.5 Mb of *B. napus* chromosome A03, which included a fragment of the markers DM_A03_10873502 and DM_A03_12570715 for *Rcr10^ECD01^*, was homologous to the region 11.0–16 Mb of “Chiifu” A03 (Figure 5). *Rcr9^ECD01^*, located on the 2.2 Mb length from 9.3 to 11.5 Mb of *B. napus* chromosome A08, which included SNP markers DM_A08_10325589 and DM_A08_10529713, was homologous to the region 12.0–14.5 Mb of A08 in *B. rapa* “Chiifu” (Figure 5).

## Discussion

Clubroot has the potential to become an important constraint to canola production on the Canadian Prairies. Pathotype 3H was (and likely still is) the predominant pathotype in the Prairie region, pathotype 5X was the first new pathotype identified as virulent on resistant canola cultivars such as “45H29,” and 3A and 3D have become the most prevalent among the new virulent pathotypes (Hollman et al., 2021). Therefore, these four pathotypes were selected for this study.

Two QTLs for resistance to the four pathotypes of *P. brassicae* derived from *B. rapa* ECD01 were transferred to, identified, and mapped in a DH population of *B. napus*. The DH population was segregated in a 1:3 (R:S) ratio for resistance to pathotype 3A. This indicated that resistance to pathotype 3A was controlled by two genes in complementary action. The segregation ratio for resistance to pathotype 3H was 3:1, which was also the most likely fit for pathotypes 3D and 5X. This indicated that resistance to all three pathotypes in the DH population was controlled by two genes in duplicate action. Two QTLs, *Rcr10^ECD01^* and *Rcr9^ECD01^*, for resistance to pathotypes 3A, 3D, and 3H were identified, which was consistent with the genetic analysis of phenotype ratios. However, only one QTL, *Rcr9^ECD01^*, was identified for resistance to 5X, although the segregation ratio was close to 3:1. This inconsistency merits further investigation.

In general, strong resistance to clubroot pathotypes is controlled by single dominant genes such as *Rcr1*–*Rcr7* (Chu et al., 2014; Yu et al., 2016; Huang et al., 2017, 2019; Yu et al., 2017; Dakouri et al., 2018; Chang et al., 2019; Karim et al., 2020). Two genes in duplicate action (*Rcr8* on chromosome A02 and *Rcr9* on chromosome A08 from *B. rapa* line T19) that conferred resistance to pathotype 5X were reported previously (Yu et al., 2017). Similarly, a previous study indicated that neither *Crr1* nor *Crr2* on their own conferred resistance to Japanese strain “Wakayama-01” of *P. brassica*; resistance was only expressed when resistance alleles were present at both loci (Suwabe et al., 2003). In this study, the QTL for resistance to pathotype 3A derived from ECD01 may behave similarly to *Crr1* and *Crr2.*

The number of SNP sites per chromosome is usually correlated with chromosome size in mapping populations (Yu et al., 2016) but was not correlated in this study. This unusual result likely occurred because the BC_2_ donor plant used for microspore culture carried a large fragment of chromosome A01 originating from ECD01 but smaller fragments of the other chromosomes from ECD01.

In this study, the target region for *Rcr10^ECD01^* was defined as 9.8–13.5 Mb of *B. napus* chromosome A03 using QTL analysis. A similar interval (9–14 Mb) for *Rcr10^ECD01^* was obtained using the identification of the PPV with BSA. The region for *Rcr10^ECD01^* in *B. napus* was homologous to the 11.0–16.0 Mb region of A03 in the *B. rapa* “Chiifu” version 3.0. This was a distinct genetic region from *Rcr1*, *Rcr2*, *Rcr4*, and *Rcr5* for resistance to pathotypes of *P. brassicae* (Figure 4). The genes *Rcr1*, *Rcr2*, and *Rcr4*, which confer resistance to pathotypes of *P. brassica*, have previously been mapped into chromosome A03 of *B. rapa* “Chiifu” version 3.0 at ∼25 Mb region (Chu et al., 2014; Yu et al., 2016; Huang et al., 2017), while *Rcr5* was also mapped at ∼24 Mb region in that chromosome (Huang et al., 2019; Figure 4). *Rcr1*, *Rcr2*, and *Rcr4* were subsequently co-localized with the cloned CR genes *CRa/CRb*^kato^** (Ueno et al., 2012; Hatakeyama et al., 2017), while *Rcr5* was located in a region close to *CRa/CRb*^kato^**. In addition, resistance genes *Rcr1*, *Rcr2*, *Rcr4*, and *Rcr5* were identified for resistance to pathotype 3H, not for 3A, 3D, or 5X. Several CR genes or QTLs, such as *PbBa3.2* (Chen et al., 2013), *CRd* (Pang et al., 2018), *Crr3* (Hirai et al., 2004), and *CRk* (Sakamoto et al., 2008) for resistance to clubroot strains collected from Japan and China, have been mapped into the regions different from *CRa/CRb*^kato^**. Similarly, *BraA.CR.c* for resistance was mapped into chromosome A03 in turnip cvs. ECD01, ECD02, and ECD04 (Hirani et al., 2018). The relationship of *Rcr10^ECD01^* to these previously identified genes needs to be determined. Also, *CRb* was identified in a Chinese cabbage cv. “CR Shinki” was originally derived from ECD01 for resistance to *P. brassica* strains collected in South Korea (Piao et al., 2004). It was located in a genetic region close to *CRa/CRb*^kato^**. However, no QTL in the *CRb* region was identified in this study.

A QTL, identified and designated as *Rcr9^ECD01^* (because it was mapped into the genetic region of *Rcr9* and was originally derived from *B. rapa* cv. ECD01), conferred resistance to all four pathotypes (3A, 3D, 3H, and 5X) assessed in this study. *Rcr9^ECD01^* was located on the 2.2 Mb length from 9.3 to 11.5 Mb of *B. napus* chromosome A08 using QTL analysis. The *Rcr9^ECD01^* interval was confirmed through the identification of PPV with BSA, located in the physical interval 9–12 Mb. The region of *Rcr9^ECD01^* in *B. napus* corresponded to 12.0–14.5 Mb of A08 in *B. rapa* “Chiifu” version 3.0 (Figure 4).

Previously, our laboratory had identified *Rcr9* for resistance to pathotype 5X in *B. rapa* breeding line T19, which originated from German turnip cv. “Pluto” (Yu et al., 2017). The proposed position of *Rcr9* spanned a large interval (6.48 Mb) of chromosome A08, including the genome region of *Rcr3* and *Rcr9^ECD01^*. However, several breeding lines that carried *Rcr9* were resistant to 5X, but not to 3A, 3D (Yu, unpublished), and 3H (Yu et al., 2017). This difference in phenotype indicated that *Rcr9* differed from *Rcr9^ECD01^*. Another resistance gene, designated as *Rcr9^wa^*, has also been identified from a turnip differential line in the ECD. It originated from cv. “Waaslander” (ECD04), provided resistance to pathotype 5X, and was mapped into the same region as *Rcr9* (Karim et al., 2020). *Rcr9^wa^* was mapped based on flanking markers into 12.3–12.6 Mb of chromosome A08 (smaller interval than *Rcr9*). In addition, another resistance gene originated from cv. “Waaslander” and conferred resistance to pathotype 3H, designated as *Rcr3*, has been mapped into chromosome A08, flanked by SNP markers in position 11.3–11.6 Mb in the *B. rapa* “Chiifu” reference genome version 3.0 (Karim et al., 2020). The position of *Rcr3* was separated from *Rcr9^ECD01^* (Figure 4). Also, gene *BraA.CR.b* for resistance to pathotype 3H was previously identified from the turnip differentials ECD01, ECD02, ECD03, and ECD04 and mapped into chromosome A08 (Hirani et al., 2018), but no information on the genome region corresponding to the *B. rapa* “Chiifu” reference genome version 3.0 was provided. Several genes for resistance to collections of *P. brassicae* from Japan and China, including *Crr1* (Suwabe et al., 2003), *CRs* (Laila et al., 2019), *PbBa8.1* (Chen et al., 2013), and *qBrCR38-2* (Zhu et al., 2019), have also been mapped into chromosome A08. The cloned CR gene *Crr1* was highly homologous to *Bra020861* in the *B. rapa* reference genome version 1.5 and to *BraA08g014480* in the *B. rapa* reference genome version 3.0, which is located in the *Rcr9^ECD01^* genomic region. However, breeding lines carrying *Crr1* gene did not show resistance to the strains of *P. brassica* used in this study (Yu, unpublished). Therefore, *Rcr9^ECD01^* is unlikely the same as *Crr1*. The relationship of *Rcr9^ECD01^* with *CRs* (Laila et al., 2019), *PbBa8.1* (Chen et al., 2013), and *qBrCR38-2* needs to be determined.

*CRc* was identified in the Chinese cabbage cv. “CR Kanko” derived from “Debra,” which was located into chromosome A02 (Sakamoto et al., 2008). However, this gene was not found in the DH population used for this study.

Analysis of QTLs has been used for the identification of several major genes for resistance to clubroot (Yu et al., 2017). A QTL that can be consistently detected with a PVE of >10% of trait value can be designated as the main effect QTL or major QTL (Wang et al., 2019). In this study, QTLs *Rcr10^ECD01^* and *Rcr9^ECD01^* were identified with 16.2 to 43.3% PVE. *Rcr10^ECD01^* was identified based on the response to inoculation with pathotypes 3A, 3D, and 3H. *Rcr9^ECD01^* was identified based on the response to inoculation with all of the pathotypes used in this study. Therefore, both *Rcr10^ECD01^* and *Rcr9^ECD01^* appear to be major QTLs. The presence of the two major QTLs was also confirmed through BSA, which is consistent with the result obtained from the QTL analysis.

Clubroot severity in the DH lines in response to inoculation with the individual pathotypes was highly correlated, which indicated that resistance to these pathotypes was likely controlled by the same gene or tightly linked genes. However, the identification of QTLs in this study was based on relatively rough gene mapping, so it could not be determined if resistance to the pathotypes was controlled by a single gene or tightly linked genes. More detailed studies are in progress.

## Data Availability Statement

The original contributions presented in the study are included in the article/Supplementary Material, further inquiries can be directed to the corresponding author.

## Author Contributions

FY conceived the study, designed the experiments, performed the data analysis, and drafted the manuscript. YZ and JW developed the population, performed the data analysis, and collected phenotypic data. QC and MK performed the data analysis. BG and GP provided important materials. All authors reviewed the manuscript and approved the final draft.

## Conflict of Interest

The authors declare that the research was conducted in the absence of any commercial or financial relationships that could be construed as a potential conflict of interest.

## Publisher’s Note

All claims expressed in this article are solely those of the authors and do not necessarily represent those of their affiliated organizations, or those of the publisher, the editors and the reviewers. Any product that may be evaluated in this article, or claim that may be made by its manufacturer, is not guaranteed or endorsed by the publisher.

## Footnotes

1
https://www.genoscope.cns.fr/brassicanapus/data/

## Supplementary Material

Supplementary Figure 1 The total numbers of sequences obtained from genotype by sequencing and the number of sequence aligned into the A-genome of *B. napus* “Darmor-*bzh*” version 4.1 for each line in the DH population.

Supplementary Figure 2 The linkage map of A-genome chromosomes of *B. napus* developed from 260 SNP sites. Note that the A06 chromosome did not contain any SNPs and so is not represented.
