# Supplementary figures and images for: Identification of Two Major QTLs in Brassica napus Lines With Introgressed Clubroot Resistance From Turnip Cultivar ECD01
Source: Front Plant Sci. 2022 Jan 12;12:785989. doi: 10.3389/fpls.2021.785989 (PMC8790046; doi:10.3389/fpls.2021.785989)

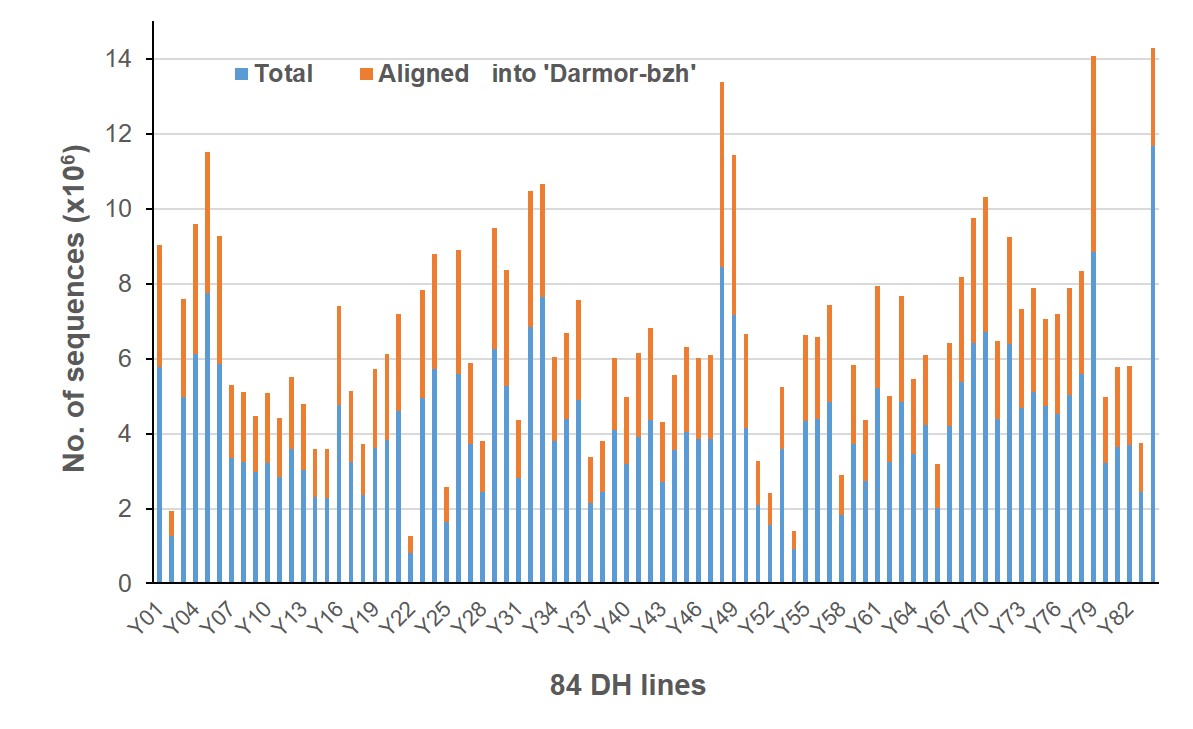

Supplement: Supplementary Figure 1 — The total numbers of sequences obtained from genotype by sequencing and the number of sequence aligned into the A-genome of B. napus “Darmor-bzh” version 4.1 for each line in the DH population. [file Image_1.JPEG]

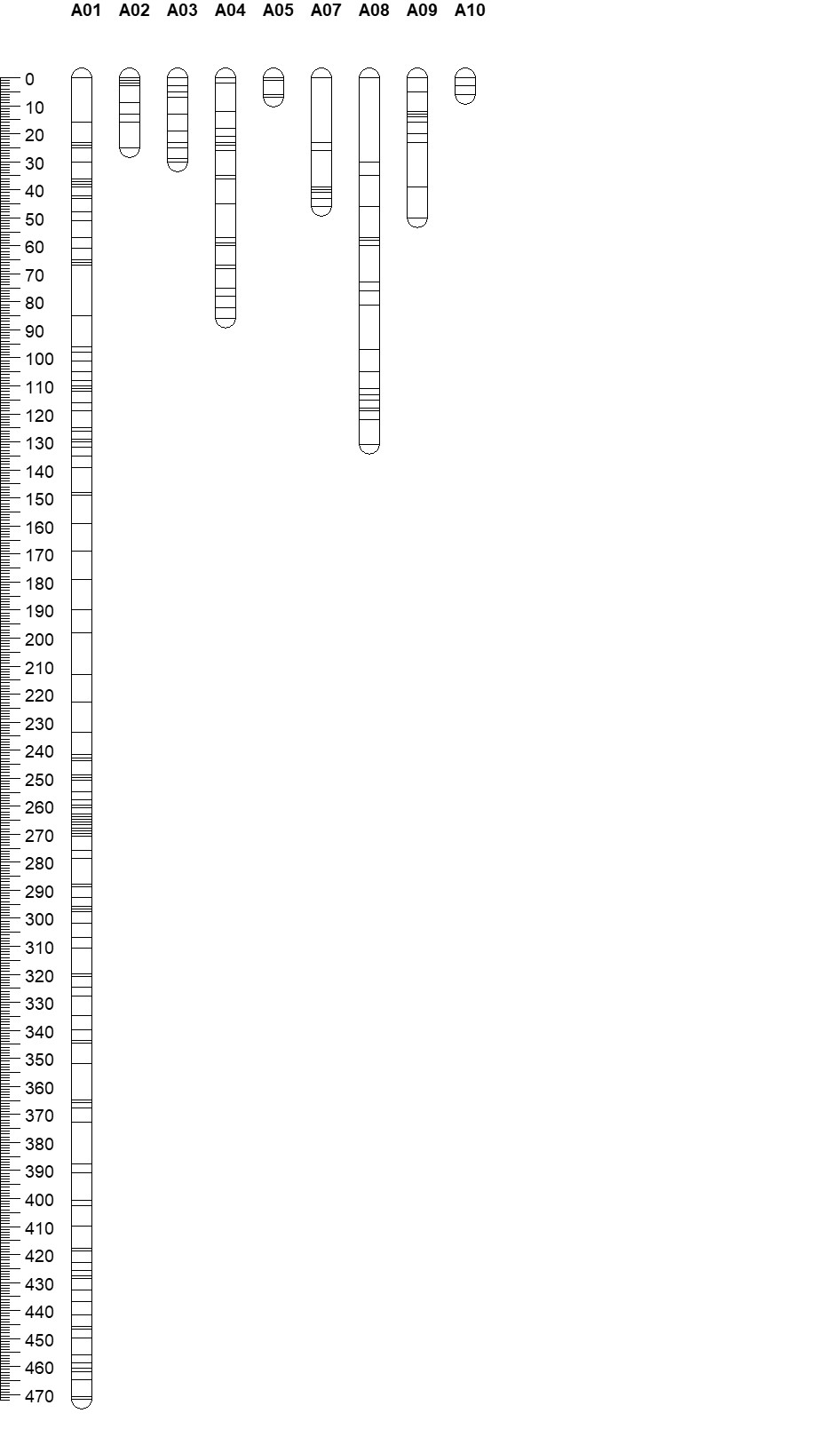

Supplement: Supplementary Figure 2 — The linkage map of A-genome chromosomes of B. napus developed from 260 SNP sites. Note that the A06 chromosome did not contain any SNPs and so is not represented. [file Image_2.JPEG]
